# Predicting Response to Preoperative Chemotherapy Agents by Identifying Drug Action on Modeled MicroRNA Regulation Networks

**DOI:** 10.1371/journal.pone.0098140

**Published:** 2014-05-21

**Authors:** Lida Zhu, Juan Liu, Fengji Liang, Simon Rayner, Jianghui Xiong

**Affiliations:** 1 School of Computer Science, Wuhan University, Wuhan, P. R. China; 2 State Key Lab of Space Medicine Fundamentals and Application (SMFA), China Astronaut Research and Training Center (ACC), Beijing, P. R. China; 3 Key Laboratory of Agricultural and Environmental Microbiology, Wuhan Institute of Virology, Wuhan, China; 4 The CUHK-ACC Space Medicine Centre on Health Maintenance of Musculoskeletal System, The Chinese University of Hong Kong Shenzhen Research Institute, Shenzhen, China; University of Erlangen-Nuremberg, Germany

## Abstract

Identifying patients most responsive to specific chemotherapy agents in neoadjuvant settings can help to maximize the benefits of treatment and minimize unnecessary side effects. Metagene approaches that predict response based on gene expression signatures derived from an associative analysis of clinical data can identify chance associations caused by the heterogeneity of a tumor, leading to reproducibility issues in independent validations. In this study, to incorporate information from drug mechanisms of action, we explore the potential of microRNA regulation networks as a new feature space for identifying predictive markers. We introduce a measure we term the CoMi (Context-specific-miRNA-regulation) pattern to represent a descriptive feature of the miRNA regulation network in the transcriptome. We examine whether the modifications to the CoMi pattern on specific biological processes are a useful representation of drug action by predicting the response to neoadjuvant Paclitaxel treatment in breast cancer and show that the drug counteracts the CoMi network dysregulation induced by tumorigenesis. We then generate a quantitative testbed to investigate the ability of the CoMi pattern to distinguish FDA approved breast cancer drugs from other FDA approved drugs not related to breast cancer. We also compare the ability of the CoMi and metagene methods to predict response to neoadjuvant Paclitaxel treatment in clinical cohorts. We find the CoMi method outperforms the metagene method, achieving area under curve (AUC) values of 0.78 and 0.66 respectively. Furthermore, several of the predicted CoMi features highlight the network-based mechanism of drug resistance. Thus, our study suggests that explicitly modeling the drug action using network biology provides a promising approach for predictive marker discovery.

## Introduction

Discovering predictive markers in cancer treatment remains a priority as identifying patients most responsive to specific chemotherapy agents helps to maximize the benefit of treatment and to minimize unnecessary side effects. Several predictive biomarkers have been identified based on gene expression data using associative learning strategies which are primarily derived by using independent gene expression values as predictive features [1], [2], [3]. The genomic study of larger collections represents a novel approach for predictive marker discovery by identifying such markers in the preclinical setting and then validating them in a clinical trial [4], [5]. Furthermore, determining drug resistance mechanisms can aid the development of treatment strategies to overcome existing limitations in therapeutic efficacy. However, the effectiveness of such approaches can be limited by the biological heterogeneity that exists within tumors which can produce predictive gene expression signatures that are simply a consequence of chance associations, leading to impaired reproducibility in independent validations [6], [7]. There is accumulating evidence that identifying drug response pathways can help to predict treatment sensitivity [8], [9]. In such approaches, rather than performing a gene-by-gene associative analysis, distinct pathways within gene expression datasets are quantified based on mRNA values or by defining metagenes according to functional RNA interference analysis of drug response pathways [3], [6]. The Connectivity Map [10] (CMAP) was one of the first attempts to perform large-scale identification of novel drug indications and investigated gene expression changes within a preclinical setting to highlight how these signatures can reflect the complex interactions between small molecules, genes and disease. The study identified drug response profiles and searched for the negative correlations between disease expression and treatment expression signatures to construct an in silico drug screening tool.

However, faced with the underlying complexity of drug induced perturbations in heterogeneous tumor tissues, we hypothesis that the development of ideal predictive markers should also incorporate information from the drug action mechanisms. MiRNAs have been identified to play a major role in a wide range of key cellular processes such as growth, development and apoptosis [11], [12], [13] and they are predicted to regulate up to one third of all protein-coding genes [14]. There is widespread evidence that microRNAs can act as tumor suppressors or oncogenes and their dysregulation is broadly associated with cancer initiation and progression [15], [16]. There is now multiple evidence that microRNAs are key regulators in highly connected multi-level transcriptome regulation networks (e.g. [17], [18]) as well as data highlighting the role of microRNAs in defining cancer related phenotypes such as prognosis [19], [20], [21], [22], [23], [24], [25] and their role as key mediators of drug actions [17], [18], [26], [27], [28]. However, compared to gene expression data, there are still limited numbers of comprehensive miRNA expression data available. Therefore, as an alternative, we consider here the microRNA regulation network an alternative feature space to interrogate drug action.

**Figure 1:**
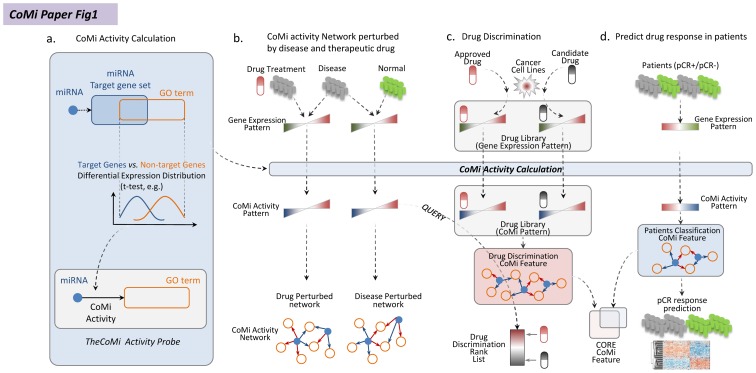
The overall framework of the present study. (a.) The CoMi method calculation process. The CoMi pattern is formed by integrating gene expression data with miRNA target and Gene Ontology information. (b.) Based on the disease differential data, we generate a CoMi disease induced network. For the drug perturbation case, we can also generate a CoMi network to represent the perturbed drug mechanism of action. In order to find the treatment for the disease, we compared the negative correlation between the cancer-induced CoMi patterns and drug treatment induced CoMi patterns. (c.) From the drug perturbation data, we build a library of drug-perturbed CoMi networks. The CoMi network can then be trained to discriminate FDA-approved breast cancer drugs from other FDA approved treatments not associated with breast cancer. (d.) Application and evaluation of the ability of the CoMi method to predict response to drug treatment from clinical data. By comparison of the drug discrimination CoMi feature with the patient response prediction CoMi patterns under similar treatment, the drug's mechanism of action can be interrogated.

We propose that investigating miRNA regulation on specific regions of the transcriptome might provide a novel perspective for relating disease state to drug treatment action. The framework of our study is shown in Fig 1 (See Methods for details). Consistent with experimental evidence, we assume that a microRNA regulates a functional gene set by targeting its target gene within the gene set and consequently impacting their expression. To this end, we introduce the concept of the Context-specific MiRNA (or CoMi) to provide a measure of the effect of miRNA regulation on a specific gene set. We achieve this by calculating the statistical difference between the distributions of target and non-target gene expression for a specific miRNA, where the gene set is determined from “specific context” data. This “specific context” can be defined from different perspectives, such as signaling pathway data, protein-protein-interaction (PPI) networks [29], [30] and Gene Ontology [31]. Thus, a set of genes with a “specific context” can be tagged with a specific biological function background, for example, sharing a common regulatory mechanism. Using this approach, we systematically evaluate the potential of the CoMi feature space for representing the drug mechanism of action and then demonstrate how the CoMi features can be used to predict patient response to Paclitaxel in three clinical trials.

## Results

### Generation of CoMi pattern networks for Methotrexate treatment in Breast Cancer

**Figure 2:**
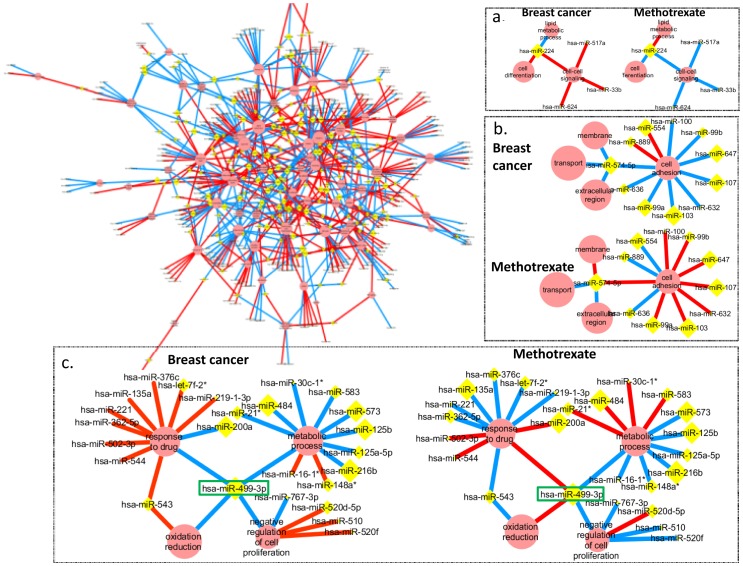
The background newtork is the complete network generated for differential breast cancer data. And three examples of identified cancer associated CoMi sub-networks and their corresponding drug induced CoMi networks. The GO term nodes are represented by red circles and miRNA nodes by yellow diamonds. The figure also shows specific sub-networks for associated with breast cancer (left sub-network) and treatment with Methotrexate (right sub-network) for (a) “cell-cell signaling”, (b) “cell adhesion” and (c) “response to drug/metabolic process/negative regulation of cell proliferation”. The node size is proportional to the degree of the node. The value of the edge of CoMi pattern pair is the CoMi Index and the width of the edge represents the value of -log10(P value). The red edges indicate that the target genes of miRNAs associated with the indicated GO term are down-regulated. Conversely, blue edges indicate the target genes are up-regulated. In each of these three cases the regulation pattern of the breast cancer network is inversely correlated with the regulation pattern for the Methotrexate sub-network.

We first generated the CoMi network for breast cancer in the absence of treatment (i.e. cancer vs. normal) from gene expressions sets for 43 paired patients and extracted the most significant CoMi patterns which represented the differential expressed disease signature (P-value<0.05). The network is composed of a series of edges connecting a miRNA node to a node representing a specific GO term (Fig 2) which corresponds to the CoMi pattern. Since a specific miRNA can regulate many mRNAs, which in turn are associated with other GO terms, one miRNA can have multiple edges. Conversely, a specific GO term can be affected by several miRNAs. Thus, the final CoMi pattern is a network constructed of interconnected GO terms and miRNAs.

**Figure 3:**
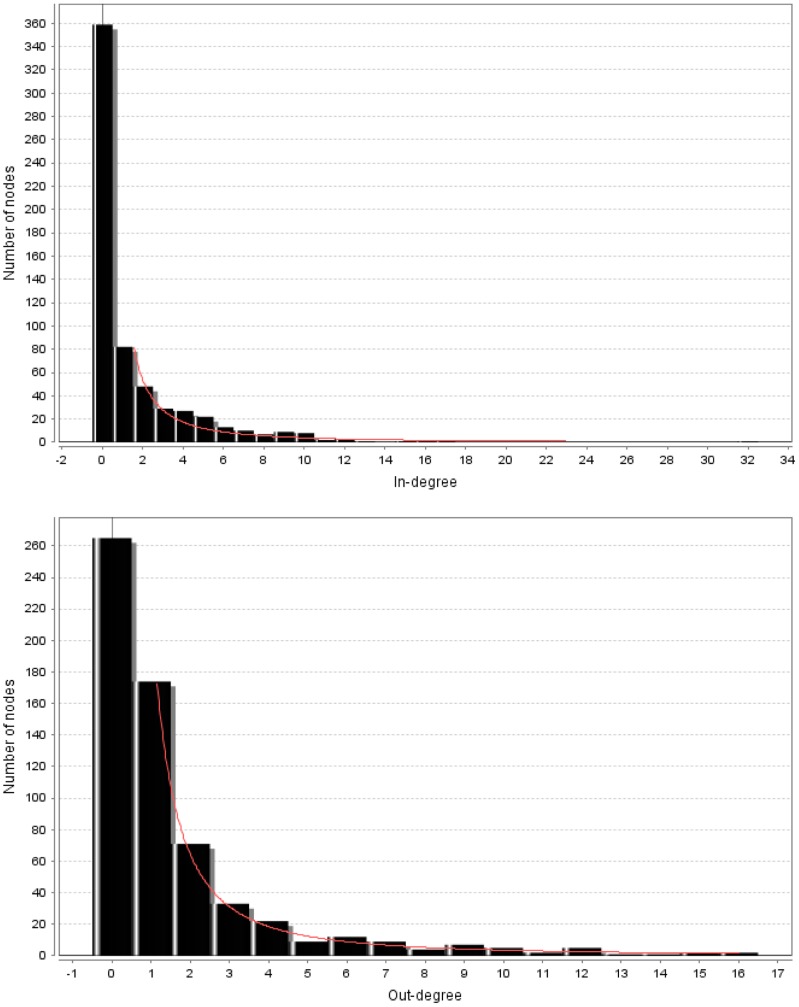
The topology of the complete CoMi network analysis of the in-degree (GO term) and out-degree (miRNA) nodes of the network fit a power law distribution. The Y-axis represents the number of nodes, the X-axis is the number of In-degree/Out-degree nodes. These graphs indicate that the generated networks are typical scale-free networks.

To generate the differential disease network, we selected the CoMi patterns which had significant changes between Cancer vs. Normal, resulting in a differential disease network of 1527 edges. Investigation of the topology of the complete CoMi network reveals that both the in-degree (GO term) and the out-degree (miRNA) nodes of the network fit a power law distribution, suggesting that the network is a typical scale-free network as shown in Fig 3. Table 1 lists several highly-connected GO term nodes with a high in-degree within the network including “Signal Transduction”, “Transport”, and “Apoptosis”. The highly-connected miRNA nodes (i.e. nodes connected to many GO terms) also highlight several cancer related miRNAs, such as the previously identified onco-miRNA hsa-miR-34a, and marker miRNA hsa-miR-183-3p [32].

**Table 1:** The most connected GO nodes and miRNA nodes in the estimated breast cancer differential CoMi network.

Degree	GO term node	Degree	miRNA node
26	signal transduction	7	hsa-miR-183-3p
25	Transport	6	hsa-miR-34a
24	Apoptosis	6	hsa-miR-103
21	protein amino acid phosphorylation	6	hsa-miR-339-3p
20	extracellular region	5	hsa-miR-892b
20	cell cycle	5	hsa-miR-216b
11	regulation of apoptosis	3	hsa-miR-27a

Fig 2 shows three examples of GO/miRNA sub-networks (i.e. local networks formed by the nearest neighbor nodes for specific nodes) for GO terms “response to drug”, “metabolic process”, “transport” and miRNAs “hsa-miR-499-3p”, “hsa-miR-574-5p”, “hsa-miR-224”. These miRNAs have already been found to be associated with breast cancer risk [33], [34], [35], [36], [37]. For the edges around the GO term node “response to drug” the CoMi indexes are all up-regulated, whereas most of the edges around the GO term node “metabolic process” are down-regulated. Thus, the CoMi network can highlight miRNAs and biological processes associated with mechanisms of cancer progression.

We then considered the perturbation pattern induced by the chemotherapy drug Methotrexate on the same CoMi sub-network based on drug induced gene expression profiles from the Connectivity Map (CMAP) dataset [10]. The sub-networks corresponding to the cancer vs. normal networks for Fig 2a are shown in Fig 2b and show striking perturbations in their regulation patterns. In the GO term “response to drug” sub-network, all the up-regulated edges in cancer vs. normal are down-regulated by Methotrexate treatment. Similar modifications to regulation are shown in the corresponding “metabolic process” and “negative regulation of cell proliferation” sub-networks and were also found in many other sub-networks within the generated CoMi network. These network changes indicate there are several miRNAs that may play pivotal roles both in breast cancer pathology and in drug action. For example, for NBN (a breast cancer suppressor), one target gene of the key regulator hsa-miR-499-3p has also been reported to be associated with increased risk of breast cancer [38], [39], [40]. However, the global impact of hsa-miR-499-3p on the transcriptome is still unknown. In Fig 2c the CoMi network associates hsa-miR-499-3p with regulation of “response to drug” and “oxidation reduction” as well as several other predicted functions. These results suggest that the CoMi pattern network can provide insight into the global impact and mechanism of drug induced transcriptome remodeling in cancer.

### Identifying successful drugs using CoMi signatures

We next investigated whether we could establish a more quantitative measure of the relationship between disease (breast cancer) and drug response (breast cancer treatments) using the CoMi network. Using drug response pattern entries based on gene expression data available from the CMAP database [10] we defined a discriminatory measure according to the following three steps. (1) Starting with a query profile (i.e., cancer vs. normal gene expression profiles), the cancer-specific CoMi activities pattern is represented as an ‘input CoMi signature’. (2) Within each query, the drug-induced CoMi network generated from the CMAP data is searched to identify drugs with signatures inversely correlated with the ‘input CoMi signature’ based on the Spearman Coefficient. (3) A ranked list of drugs is ordered according to the Spearman Coefficient. By generating a list comprised of both standard breast cancer drugs and other randomly selected drugs associated with other treatments, we can evaluate the ability of this method to identify real drugs by considering the ranking of each candidate drug.

Since several of the drugs entries in CMAP are represented by multiple instances (i.e., different concentrations and multiple cell lines), our selected dataset contains 17 drugs with different concentrations in 5 kinds of cell lines, corresponding to 103 drug instances, of which 23 were associated with FDA-approved breast cancer treatment. To investigate whether the framework could recognize these 23 breast cancer treatment instances by awarding them a high rank in the ordered drug list, a drug screening performance index (DSP index) (See Methods) and a corresponding P-value were used to estimate the reliability of the prediction. The DSP index provides a measure of the similarity of the ranked positions to the order of the original list; a positive DSP index indicates the real drugs are ranked at the top of the list. A P-value <0.05 was considered significant. We then repeated the calculation using the mRNA and CMAP data to compare the discriminatory abilities of the different three methods. The CMAP method was consistent with the method used in the CMAP web-online tools [10]. Specifically, we applied the CMAP method to our testbed to assess its predictive power in identifying 17 breast cancer treatments among 6100 instances. The mRNA based method was conducted in a similar way based on the Pearson correlation coefficient and the CoMi framework but using gene expression as the feature rather than the CoMi Index.

**Figure 4:**
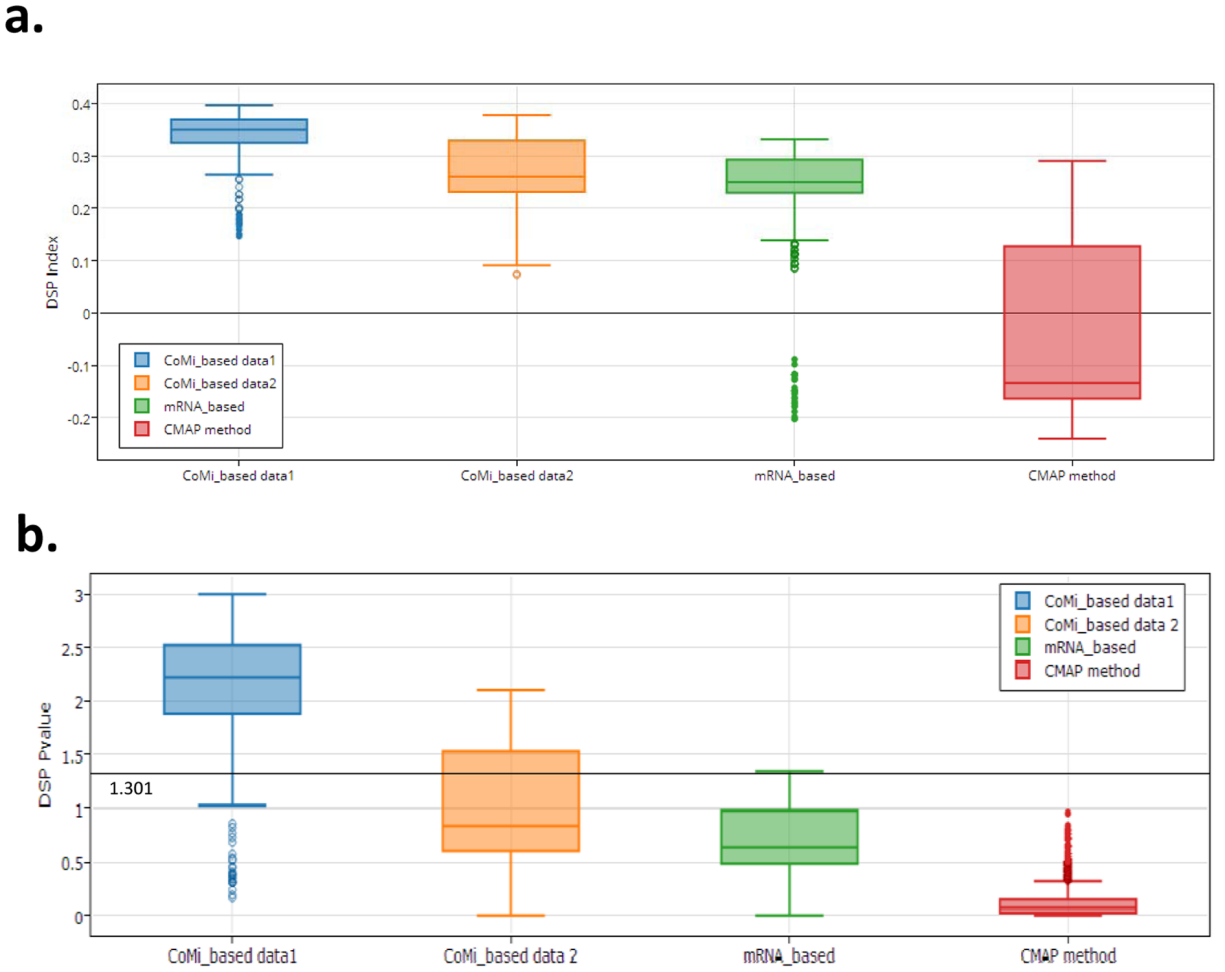
Comparison of the drug discriminative ability of three different data sources. (a.) From left to right. Drug Screening Performance (DSP) index distribution for CoMi1&2 (GSE15852, GSE5364), mRNA expression data and CMAP. The blue box indicates the CoMi method performed with GSE15852, the line within each of the box marks the median of the values. The circles indicate outliers (defined as those laying outside approximately 99.3% coverage, the default setting in Matlab). Higher DSP values indicate stronger agreement with an ordered drug list that places breast cancer treatments at the top. (b.) Corresponding estimated P-values for the three method plotted as −log10 (P-value). The horizontal blue line indicates the −log10 (0.05) threshold which is considered significant. Both of the CoMi results have all values greater than zero. Although the mRNA data has majority of points greater than zero, the majority of P-values are >0.05. DSP values for CMAP are almost equally distributed between positive and negative values and consequently, none of the associated P values are <0.05.

Fig 4 shows the results for the three different data types. The figure shows that the CoMi-based method provides better discrimination than the other two methods with the highest average DSP index and the narrowest 75% confidence interval. The CoMi method was performed from two different disease sources. Although the DSP index is greater than zero for the mRNA data, the majority of P-values are >0.05. Most of the values of DSP index for the CMAP method are less than zero, indicating the method failed to correctly identify the majority of true treatment drugs. The difference in the discriminative ability of these two methods is also highlighted in Table 2. Whereas the top of the ordered list generated using the CoMi-based method is enriched for breast cancer drugs, the same drugs are randomly distributed throughout the corresponding CMAP generated list. Each DSP index represents the performance of one input signature where the signature was selected according to a ranked list of CoMi index. The difference amongst signatures is based on the different window size used to generate the up and down-tags. So, when a poor result is obtained for a specific DSP index, this means the method is very sensitive to the length of the signature, which in turn is a consequence of sensitivity of the tags to the window size. Thus, the low robustness of the calculated DSP index for the original CMAP method suggests that CMAP is very sensitive to the input signature [41]. For example, for the patients in GSE15852, since each DSP index is associated with a corresponding input CoMi signature, we collected 666 CoMi patterns which were associated with the highest ranked DSP indices for the positive drugs as these CoMi patterns are the most discriminative for breast cancer treatment.

**Table 2:** Ranked drug list for CoMi based and CMAP based methods.

Drug list of CoMi index-based method			Drug list of CMAP method		
Rank	Drug	KS Score	Rank	Drug	KS Score
1	mitoxantrone	0.699029	1	decitabine	0.6893
2	mercaptopurine	0.572816	2	lomustine	0.4587
3	doxorubicin	0.524272	3	tamoxifen	0.4397
4	daunorubicin	0.504854	4	procarbazine	0.4369
5	lomustine	0.495146	5	chlorambucil	0.4223
6	paclitaxel	0.393204	6	mitoxantrone	0.3883
7	vinblastine	0.385113	7	paclitaxel	0.3576
8	tamoxifen	0.37448	8	etoposide	0.2646
9	azacitidine	0.288026	9	sirolimus	0.1534
10	tetrandrine	0.240291	10	daunorubicin	−0.3811
11	methotrexate	0.213592	11	tetrandrine	−0.4393
12	hycanthone	−0.16505	12	methotrexate	−0.4660
13	sirolimus	−0.24782	13	vinblastine	−0.4919
14	procarbazine	−0.25243	14	hycanthone	−0.4951
15	chlorambucil	−0.37136	15	doxorubicin	−0.5146
16	etoposide	−0.43204	16	azacitidine	−0.5728
17	decitabine	−0.85437	17	mercaptopurine	−0.9417

### Use CoMi to predict drug response in patients

Investigation of the relationship between the CoMi pattern and drug discrimination suggests that the CoMi method could provide useful insight into the mechanism of drug action for breast cancer treatments such as Paclitaxel and Doxorubicin. Therefore, we next considered the ability of the CoMi method to predict response to drug treatment in a drug treatment regime in breast cancer patients. We selected a dataset from a breast cancer cohort study [42] and identified potential predictive biomarkers for response to treatment with neoadjuvant chemotherapy. All the patients in these cohorts were treated with the T-FAC regime which is one of the most common neoadjuvant chemotherapies for breast cancer. The TFAC regime consists of Paclitaxel (T), 5-fluorouracil (F), doxorubicin (A), and cyclophosphamide(C).

To investigate the CoMi index's predictive ability for the outcome of the drug treatment process, we generated the CoMi index and their associated P-values based on the microarray data provided by three separated neo-adjuvant breast cancer clinical trials. We split the data into training and testing components and then applied various machine learning methods. (See Methods for details). We searched for the most significant CoMi patterns features where activity among the patients was highly discriminative in terms of pathological complete response (pCR) by using the t test to calculate the statistical difference of the CoMi index between the two groups in the training set. We then used a classifier to train the features in the training set, and tested them in the testing set. In the training stage we used under-sampling since the dataset was imbalanced (pCR/no pCR = 26/152). This process of training and testing was iterated repeatedly as shown in Fig 1d. In this way we identified 27 CoMi elements as potential biomarkers which were distinct in patients with and without a pCR.

We next tested the predictive performance of CoMi markers during classification of pCR and non-pCR. The CoMi index was used as input features for three classifiers (Naive Bayes, SVM and logistic regression). The classification was performed using five-fold cross-validation within each data set and repeated 100 times. The CoMi method achieved an area under the curve (AUC) of 0.78 in dataset GSE20271. For comparison, we also tested the performance of the Paclitaxel based metagenes identified in the study by Juul et al [6], and achieved a corresponding AUC of 0.66. The CoMi method also achieved an AUC of 0.76 and 0.77 using a Naïve Bayes classifier and SVM classifier respectively compared to AUCs of 0.60 and 0.56 from the Paclitaxel metagene response as shown in the Table 3.

**Table 3:** AUC scores for three different classifiers with different types of markers for Metagene and CoMi datasets.

	Logistic model	Naïve Bayes classifier	SVM
**Metagene(12)**	0.6647	0.6000	0.5564
**CoMi(27)**	0.7870	0.7560	0.7732

**Figure 5:**
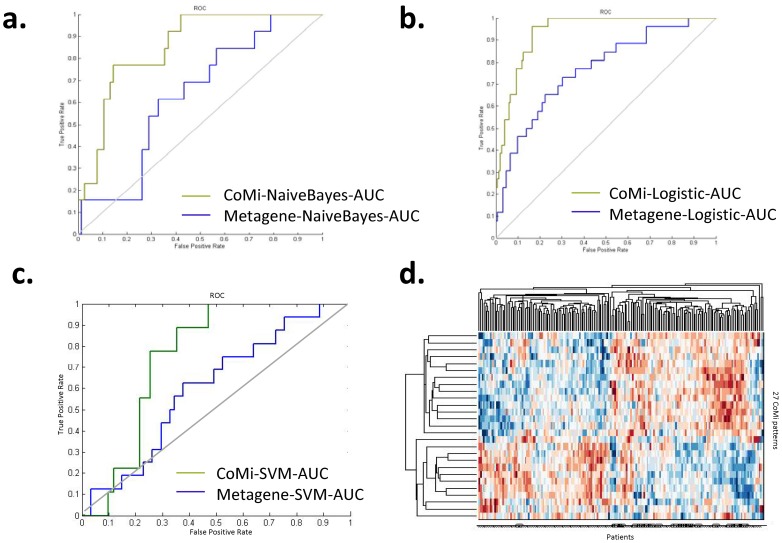
the CoMi pattern markers for pCR prediction for three different classifiers. (a) ROC using Logistic Regression (b) ROC using Naive Bayes classifier (c) ROC using SVM classifier (d) the clustergram of the identified 27 CoMi predictors (Y axis) for the outcomes of the patients response (X axis) in clinical trials MDA1+ MDA/MAQC-II. The line under the graph shows the label of the class (0 for the pCR group, 1 for the no pCR group). Most of the patients that have pCR are clustered in the right hand clade (X axis) which suggests that the markers can effectively cluster the pCR patients.

We further independently tested the potential markers in other two cohorts MDA1 and MDA/MAQC-II [2], [43], [44]. The features tested both CoMi patterns and Paclitaxel metagenes for comparison. For independent validation, we used the features identified from GSE20271 to test MDA1+ MDA/MAQC-II, and achieved an AUC of 0.78 using a logistic regression model. As shown in Fig 5a-c, the ROC of three different classifier results also highlights the improved accuracy of the CoMi method over the metagene method [6].

**Figure 6:**
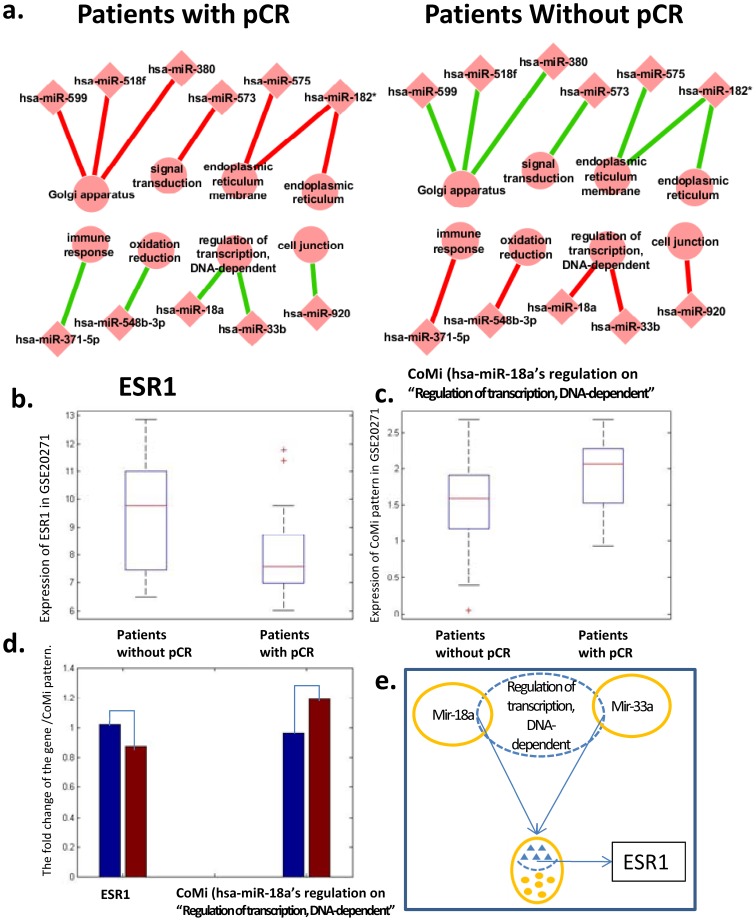
The CoMi pattern used to predict pCR in clinical trials involving Paclitaxel. (a) Graphical summary of correlation analysis between the CoMi pattern across samples and the outcome of pCR status. The edges represent activities pattern where green lines indicate highly negative correlations, red lines indicate positive correlations. (b) The expression distribution of the gene ESR1 in the two groups of pCR and no pCR patients. (c.) The distribution of the CoMi index (hsa-miR-18a on “Regulation of transcription, DNA-dependent”) in two the groups of patients. (d.) The fold change of the average expression from the patients without pCR to the group of people has pCR of the gene ESR1 and the CoMi pattern. The blue bar represents the average value of CoMi Index of patients without pCR, while the red bar is the average value of the group of patients with pCR. (e.) The regulation network of two CoMi activities with its target gene. Gene ESR1 is the target of the CoMi pattern of hsa-miR-18a's regulation on the GO term “Regulation of transcription, DNA-dependent”, it is also the target of the CoMi pattern of hsa-miR-33a's regulation on the GO term “Regulation of transcription, DNA-dependent”.

For the 27 identified CoMi features, we next investigated the correlation between the CoMi pattern across samples and the outcome of pCR status. Several CoMi markers showed a significant positive correlation with the group of patients with pCR, and a significantly negative correlation with the group of patients without pCR (Fig 6a). The CoMi pattern of potential markers in this figure also reveals several interesting regulatory relationships indicating miRNA regulation on GO Terms. Some of these GO Terms are closely related to drug effect, such as “immune response”, “signal transduction” and “G-protein coupled receptor protein signaling”. In particular, Fig 6a shows that hsa-miR-182-3p has a strong regulatory effect on several GOBP Terms including “endoplasmic reticulum” and “endoplasmic reticulum membrane”. This is consistent with a previous report that hsa-miR-182-3p is over-expressed in breast cancer [45]. Furthermore, ESR1 also has an association with another important onco-miRNA, hsa-miR-18a [46].

### Predictive CoMi features provide a network view of drug response

A recent paper investigated the mitotic and ceramide pathway as a predictor of response to chemotherapy with neoadjuvant Paclitaxel and 12 metagenes were ultimately shown to be significantly associated with response in pCR patients who have a pathological complete response (referred to as Paclitaxel metagenes hereafter) [6]. In this study we investigated the connection between the target genes revealed by our CoMi markers and the Paclitaxel metagenes. We selected the Paclitaxel metagene ESR1 (which is a target gene associated with the GO term “regulation of transcription, DNA-dependent” and the hsa-miR-18a target geneset) as an example and compared the expression of ESR1 in a group of pCR and no pCR patients respectively. As shown in Fig 6b, ESR1 is down-regulated in the pCR response compared with the no pCR response. The CoMi pattern of hsa-miR-18a regulation on the GO term “regulation of transcription, DNA-dependent” is up-regulated in the pCR response compared with the no pCR response (Fig 6c), indicating that hsa-miR-18a might target and down-regulate the expression of genes (such as ESR1) involved in the GO term “regulation of transcription, DNA-dependent”. Comparison of the statistical significance of the respective t test results for the pCR and no pCR groups suggests that the CoMi index is changed more significantly than the target gene ESR1. Since the CoMi index data cannot be directly compared to the gene expression data, we compared the rate of change for ESR1 and the CoMi pattern for “regulation of transcription, DNA-dependent”/hsa-miR-18a for the pCR group to the no pCR group using the fold change of the average expression. The results are shown in Fig 6d. The left-hand bars show the changes for ESR1, and the right paired bars show the changes for the CoMi pattern. The CoMi pattern not only achieves better discriminatory performance than the gene expression of the metagenes, but the pattern also provides important information on biological processes that are involved in the drug response. This is summarized in Fig 6e which shows that the GO term “regulation of transcription, DNA-dependent” is also regulated by another miRNA “hsa-miR-33b” suggesting it may be a key biological process in tumorigenesis.

### Predictive CoMi features are in line with published metagene

**Figure 7:**
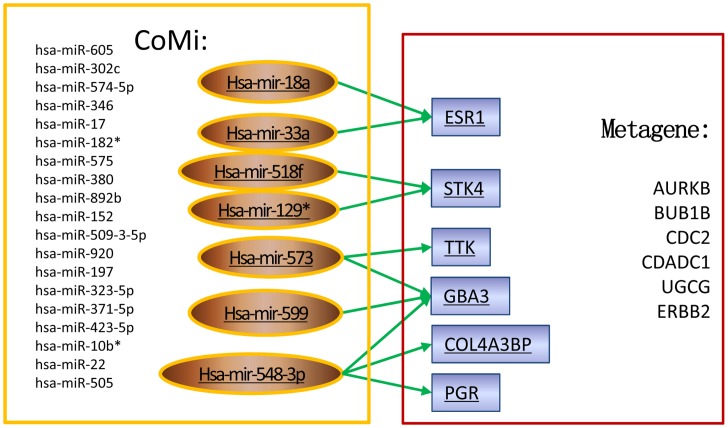
The relationship between the predictive CoMi pattern and the published metagene. The left box lists the miRNAs in the CoMi pattern that was identified as a predictive marker. The right box lists the 12 metagenes identified in the study by Juul N, et al [6] on the Paclitaxel resistant pathway. We predicted that some elements of the CoMi pattern (e.g. hsa-miR-548b-3p on the GO Term: “Oxidation reduction”) have a repressive effect on some of the metagenes (e.g. GBA3, COL4A3BP and PGR) as shown by the three arrows from the miRNA to its target genes.

Since the metagenes were selected from the Paclitaxel resistant pathway experimental study by Juul N, et al [6], their predicted CoMi features can be used to reveal their associated biological mechanisms. These are summarized in Fig 7 and Table 4. For example, from Table 4, we observe that the CoMi pattern of the effect of hsa-miR-548b-3p on the GO Term: “Oxidation reduction” has three Paclitaxel metagenes (PGR, COL4A3BP and GBA3) Paclitaxel. Interestingly, the expression of these genes didn't show significant differences between the pCR and no pCR groups (data not shown), which suggests that the CoMi feature might integrate the collective effects of marginal changes of multiple genes involved in the same GO term.

**Table 4:** List of overlapped CoMi patterns in drug-discriminating CoMi features and patients-classification CoMi features.

miRNA	GO Term
hsa-miR-302c	extracellular region
hsa-miR-574-5p	extracellular region
hsa-miR-17	mitochondrial inner membrane
hsa-miR-599	Golgi apparatus
hsa-miR-892b	plasma membrane
hsa-miR-509-3-5p	integral to membrane
hsa-miR-18a	regulation of transcription, DNA-dependent
hsa-miR-323-5p	Proteolysis
hsa-miR-22	protein transport

### Predictive CoMi features are related to drug mechanism of action

Table 4 lists the several associations between the CoMi features for Paclitaxel response and the CoMi features for breast cancer treatment discrimination. Since the patients were treated with neoadjuvant drugs, and two of these drugs, Paclitaxel and Doxorubicin, were both tested individually for their treatment effect, we next considered whether their CoMi features could show an association based on the breast cancer treatment effect. To this end, we checked the consistency of informative CoMi features in discriminating successful drugs (‘drug-classification CoMi’) with the predictive CoMi features for drug response in patients (‘patients-classification CoMi’). For the 666 drug-classification CoMi identified in section 2.2 and the 27 identified CoMi for patients' outcome there are 9 overlaps (Table 4). The significant overlap (Hyper-geometric test P-value  = 7.9226e−09) indicates that these two CoMi list are significantly consistent, suggesting these CoMis are useful not only as predictors for drug response, but also as an optimized representation of Paclitaxel action on patients.

## Discussion

In this present study we introduce the concept of the CoMi (Context-specific-MiRNA-regulation) as a representation of the miRNA regulation network in the transcriptome. One of the basic assumptions is that a miRNA is involved in a biological process through regulation of its target genes, and that a subset of genes within the same biological process are not necessarily regulated by the same miRNA. This is supported by multiple studies [12], [47], [48], [49], [50]. In this work we use the Gene Ontology (GO) to abstract a miRNA's regulation on to a specific GO biological process. In this way we can interpret the significance of an identified relationship such as cancer, normal or drug treatment.

There have been many studies that have focused on the difference between miRNA target and non-target gene expression to estimate the activity of the miRNA [51]. Here, based on the specific context definitions, a single miRNA and a particular biologically meaningful gene set, we divide the gene set into two parts consisting of the miRNA target and non-target genes. The expression differences between the two subsets are then considered to be representative of a miRNA's regulatory contribution. In this way, we define the CoMi as an abstract probe to profile the regulation intensity for each miRNA within each biologically annotated gene set.

In the present study we demonstrated the use of the CoMi as a new way to interpret the hidden biological process in the mechanism of action of drugs on the transcriptome network, and to quantitatively discriminate successful breast cancer drugs from their peers by combining miRNA target information with Gene Ontology biological annotation modules. There are already several published works that explore the miRNA's functional association through biological functional modules. Some of these approaches integrate the miRNA's target information with functional gene modules to filter the potential mRNA target and consider the potential function of miRNA [52], [53]. Another approach uses the miRNA's target information enriched in similar Gene Ontology terms to build a synergistic miRNA-miRNA network [54]. These authors have also used drug perturbation data to construct a miRNA-miRNA network in an attempt to interfere possible roles in cancer in order to identify drug candidates [55]. In our work, we are able to abstract the miRNA's regulation on a specific GO biologic process, and describe its significance and repression level. We use the CoMi as a novel perspective for analyzing breast cancer differential data and use this to build a CoMi network. Furthermore, we link the disease and drug by using the strong negative correlation of a miRNA's regulation in different conditions to discover the CoMi network of drug related function and the drug mechanism of action. This is the first report that uses such a network to analysis the drug mechanism of action and predicts drug sensitivity.

Following the pioneering work of the Connectivity Map, there have been several subsequent efforts that attempt to use gene expression signatures or modules to identify potential drug treatments [10], [41], [56]. However, these analyses are very sensitive to the input signature [41]. By incorporating gene expression data and the miRNA regulations that describe the underlying biological network, we demonstrate that our CoMi network based approach offers greater sensitivity compared to methods based only on gene expression data.

In recent years, an increasing number of disease markers have been identified through analysis of genome-wide expression profiles [42]. However, these marker sets appear to share very little in common and lack functional network insight information. This highlights one the primary limitations of using predictive gene expression signatures based on small and unbalanced samples; this leads to impaired reproducibility in independent validations [6], [7]. By integrating gene expression profiles from clinical trials with our CoMi method, the data is mapped into a CoMi pattern. We can then identify several CoMi activities with significant P-values as potential biomarkers for the response to the therapy.

Our results show that CoMi features outperform published gene expression patterns or ‘metagenes’ selected from functional RNA interference analysis of drug response pathways [6] and demonstrate that the CoMi biomarkers can also be related to the gene target in the drug response pathway. Results on the benchmark datasets indicate this approach is effective and outperforms the gene expression method, and can reveal additional informative features related to drug response mechanisms.

Nevertheless, despite these advantages, there is still room for improvement. The current limitations in accuracy and specificity of miRNA target prediction adds noise to the network, and redundancy and inaccuracies in the Gene Ontology causes further imprecision in the predicted network patterns. As these are refined through experiment and curation, this will improve further the prediction efficiency and inaccuracy.

## Materials and Methods

### Workflow

The study is organized into four parts (Fig 1): (1) Using the gene expression as input and the Gene Ontology as context, we integrate miRNA target information with associated GO terms to calculate the CoMi profile. (2) Using breast cancer as an example, we combine the disease and drug perturbation gene expression patterns with the CoMi activities and map the data into a CoMi pattern. This CoMi pattern can then be used to build a disease-specific CoMi network and a drug differential CoMi network. (3) We then establish a drug discrimination method to verify the CoMi's performance by promoting FDA approved breast cancer drugs within the library to extract the most discriminative CoMi features related to breast cancer. (4) Finally, we convert the clinical data of breast cancer treated with neoadjuvant Paclitaxel into a CoMi pattern, then predict response to neoadjuvant Paclitaxel using CoMi features, demonstrating the CoMi features reveal more informative biological meanings compared to the mRNA based method.

### Datasets used in the study and generation of target and non-target gene sets

The Breast cancer disease datasets were selected from the Gene Expression Omnibus (accession number GEO GSE15852, GSE5364) and contained expression data from 43 and 13 human breast tumors and their paired (adjacent) normal tissues respectively. Each gene in the expression dataset was mapped to a subset of Gene Ontology terms (i.e., Gene Ontology Biological Process-GOBP and Gene Ontology cellular component-GOCC) to define a “context-specific” gene set. The age range was from 22 to 79. The data were analyzed with Microarray Suite version 5.0 (MAS 5.0) and GCOS version 2.0 using the default Affymetrix analysis settings. The clinical diagnosis was limited to a few terms: infiltrating ductal carcinoma, invasive ductal carcinoma, lobular carcinoma, mucinous carcinoma and ductal carcinoma. MiRNAs target gene information was generated for each miRNA by collecting target predictions from the online miRNA target prediction sources, (ExprTargetDB [57] which combines miRanda [58], TargetScan [59] and PicTar [60]) and consolidating as described in detail in our earlier report [32].

### Generation of the CoMi Network and CoMi patterns

**Figure 8:**
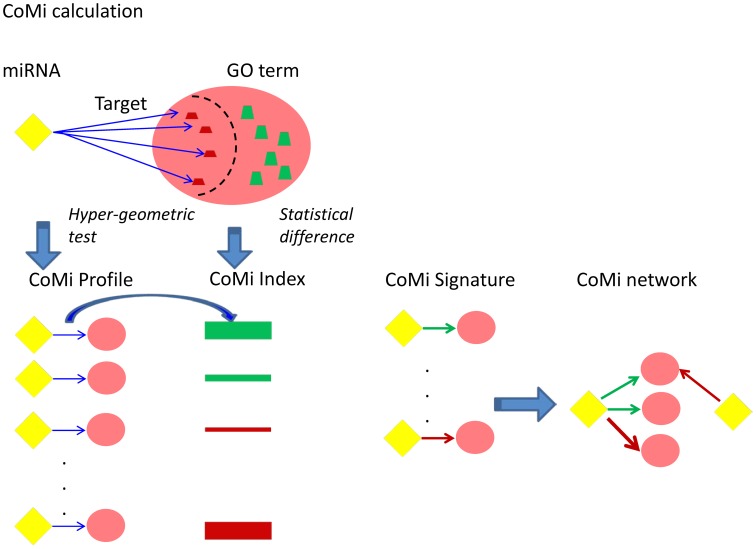
Schematic of generation of CoMi profile for a specific miRNA/GO Term pair. The yellow diamond represents a miRNA, the trapezoid represents a gene. Some of the genes could be repressed by its targeting miRNA as shown by a arrow from the miRNA to the gene. The green and red colours correspond to up- and down-regulation respectively and the height of the gene trapezoid correponds to expression level. The pink ellipse encompassing the set of genes represents a GO term. An arrow from an miRNA to a target gene within a GO term indicates this miRNA has a significant effect on this GO term as determined by the hypergeometric test and this miRNA/GO term pair is referred as a CoMi pattern. By searching each GO term for significant miRNA pairing we generate a CoMi profile and and an associated CoMi index which represents how much influence a specific miRNA influences the GO term (calculated from the statistical difference between the miRNA's target and non target genes expression within the GO term). By considering different perturbations (e.g. cancer or drug effects), we can build a CoMi network based on these CoMi patterns and by extracting the most significant CoMi patterns according to the estimated P-value of the CoMi index, we can generate breast cancer signature based on CoMi patterns and generate a CoMi network.

The CoMi network was generated based on the premise that a given miRNA, miRNAi, targets several genes represented by a gene set Mi. Similarly, a given GO term GOj (taken from the Gene Ontology Biological Process-GOBP and Gene Ontology cellular component-GOCC vocabulary) will be mapped to several genes, forming a “context specific” gene set Gj for that GO term. The intersection of these two sets Mi∩Gj can be considered to represent the regulation of GOj by miRNAi and partitions Gi into two subsets of target and non-target genes. By applying the hyper-geometric test to these target and non-target genes we can identify significant overlap for any Mi∩Gj and in these cases generate a corresponding CoMi pattern CoMiij. Taken together, the set of all CoMiij pairs represent a CoMi profile and, because of the overlap between different CoMiij, form an overall CoMi network for the miRNA/GO dataset (Fig 8).

A given CoMi pattern CoMiij does not change, i.e., the target and non-target gene sets are fixed. What can change for a disease or drug treatment is the expression levels of the genes in the target and non-target sets. We quantify these expression levels by defining a CoMi index for each condition (i.e. normal vs. disease vs. drug treatment). This CoMi network can then be used to represent (i) gene expression data from drug treatment in CMAP (DRGEXP), and (ii) disease expression data from GEO (DISEXP) by calculating a CoMi index for each CoMi pattern identified in the previous step for DRGEXP and DISEXP respectively. Significant changes between target and non-target genes for a given CoMiij were identified by using the t-test (described below) and are assumed to correspond to the effect of the up or down regulation by miRNAi on GO term GOj.

The respective CoMi profiles for DRGEXP and DISEXP can then be compared to identify significantly inversely correlated profiles. For example, if the disease state produces a significant down regulation of a group of genes by miRNAi, then the drug treatment affects a corresponding up-regulation of the same gene set. In this way, disease relevant CoMi networks can be identified.

### Generation of networks

Networks were generated using Matlab and Cytoscape was used for visualization [61]. Network topology was investigated using the Network Analysis plug-in for Cytoscape. Because miRNA are mediators, i.e., they have the ability to silence a gene, the miRNAs were considered outgoing edges and GO terms incoming edges, and the in-degree and out-degree of each node was determined.

### Identification of CoMi patterns significantly associated with disease and with drug treatment

To identify significant changes in target gene expression values brought about by the presence of a particular disease we compared the changes in expression values for target and non-target genes for a specific miRNA for normal and disease states. For each CoMi pattern CoMiij we performed the t-test as follows:
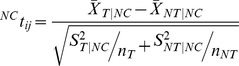
where 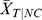
stands for the fold change vector (cancer vs. normal) of the miRNA target gene set, 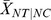
is the fold change vector (cancer vs. normal) of the non-target gene set, *n_T_* is the number of miRNAs target genes and *n_NT_* is the number of non target genes. A P-value <0.05 was considered significant.

Similarly, we identified drug induced CoMi change as follows:
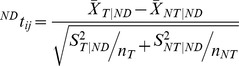
where 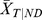
stands for the fold change vector (drug treatment vs. normal) of the miRNA target gene set, 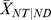
is the fold change vector (drug treatment vs. normal) of the non-target gene set, nT is the number of miRNAs target genes and nNT is the number of non target genes. A P-value <0.05 was considered significant.

The drug-induced gene expression change of known drugs was derived from the CMAP dataset, consisting of expression profiles from five different human cell lines treated with 1309 different compounds at different concentrations [10].

### Generation of the drug reference library

To build a reference library of drugs as a test dataset to investigate the ability of the CoMi method to distinguish drugs associated with disease treatment from other drugs, we selected gene expression information from the Connectivity Map [10] for a set of selected drugs and mapped them into a CoMi pattern as described above. 17 drugs were selected based on their overlap between the “standard agent database” [62] and CMAP with 103 instances in CMAP (corresponding to multiple dose treatment for one drug). 5 of these 17 were recognized breast cancer drugs in routine clinical usage (23 instances in CMAP) with successful outcome.

### Identification of CoMi patterns associated with drug treatment

To identify CoMi patterns that were associated with drug treatment we generated a disease query signature by combining the top k up-regulated CoMi activities (the up-tag), and the bottom k down-regulated CoMi activities (the down-tag) from the cancer specific CoMi patterns, where k = 5 to k = 1/3 of the number of CoMi patterns in the profile. In the same way, a query signature was calculated from each entry in the drug CoMi pattern library. Finally, the Spearman correlation coefficient was calculated between the disease query signature and each instance of the drug query and the drugs were ranked based on this value.

To determine whether this method could distinguish recognized drug treatments for breast cancer from other randomly selected drugs, all the n instances in the candidate instance library were ranked in an ascending order according to the Spearman correlation coefficient, resulting in a drug ranked list. We then used the Kolmogorov-Smirnov (KS) statistic to test whether the rank positions of the 23 successful breast cancer drug instances from CMAP were significantly enriched at the top of the ranked list.
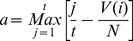





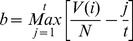





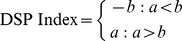



Where t the number of positive drugs in the library (23), j denotes the jth drug instance according to the rank within the N drugs in the list, and V(j) is the ranked position of the jth drug instance. To estimate the corresponding DSP P-value, the data was bootstrapped by permuting the rank position of successful-drug-related instances and counting how many times the absolute value of the random KS score was larger than the absolute value of the real KS score for 1000 times. We denoted this P-value as the “Drug Screening Performance P-value” (DSP P-value). To compare the predictive abilities of the CoMi method with alternative features we also calculated the DSP based using gene expression values based on mRNA as well as the original CMAP method, but using the Spearman coefficient rather than the KS statistic.

### Patient CoMi pattern calculation

We retrieved the gene expression and treatment response data from three cohorts from three neo-adjuvant breast cancer clinical trials. The datasets (GSE20271, MDA and MDA/MAQC-II) [2], [43], [44] with associated Affymetrix microarray analyses were derived from the primary tumor prior to drug treatment (Paclitaxel). Since the MDA1 and MDA/MAQC-II trials were done by the same investigators, at the same site, with the identical gene expression platforms, we combined the two T-FAC treated cohorts to increase statistical power for univariate logistic regression analysis as performed in the original study [63]. For the 178 patients of GSE20271 and 233 patients from MDA1 and MDA/MAQC-II, the pCR (pathological complete response) status was determined at the time of surgery.

In all cases, response to therapy defined in terms of pathologic complete response (pCR) was determined at the time of surgery. We converted the gene expression pattern of each patient into a CoMi pattern as follows:
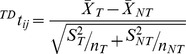
where 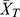
stands for the average gene expression value of the miRNA target genes, 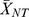
is the gene expression values of the non-target gene set, nT is the number of miRNAs target genes and nNT is the number of non target genes. A P-value <0.05 was considered significant

### Patient response prediction

To investigate the ability of the CoMi index to act as a predictor of the outcome of drug treatment process, we generated CoMi Index and their associated p-values for the datasets from the three neo-adjuvant breast cancer clinical trials above and applied various machine learning methods. The CoMi index associated P-values and clinical outcome pCR/nopCR were used as inputs. We divided the data into training data (80%) and testing data (20%) and classification was performed with five-fold cross-validation. Potential biomarker features were selected from the training set and then applied to the test set. The area under the ROC curves (AUC) for the test data was used to evaluate classification performance. The AUC value was the average value of 1000 classification results and we searched for the optimum number of CoMi features that produced the best accuracy. Three classifiers, multiple logistic regression analyses, and SVM and Naive Bayes were tested using the Matlab toolbox version 2010b.

### Comparison with identified Paclitaxel resistant metagenes

Here we compared the prediction performance of CoMi features with the metagenes based on the reported data from the work of Juul N, et al, which investigated the mitotic and ceramide pathway as a predictor of the response to chemotherapy using the neoadjuvant Paclitaxel. Twelve metagenes were identified as being significantly associated with response in pCR patients [6]. In the prediction process, the training data was generated by under-sampling to obtain a balanced dataset and the 12 metagenes were used as features to train for the Metagene dataset and then tested in the testing set repeatedly to generate an average AUC value as the same process with CoMi features. For the CoMi features that were investigated for Paclitaxel resistant, correlation between the 27 CoMi feature's Index expressions with the outcome of the pCR status is shown in Fig 6a. The Person correlation coefficient is used to estimate the relationship of the 27 CoMi and the outcome of the pCR status.
